# Equine mesenchymal stem cells from bone marrow, adipose tissue and umbilical cord: immunophenotypic characterization and differentiation potential

**DOI:** 10.1186/scrt414

**Published:** 2014-02-21

**Authors:** Danielle Jaqueta Barberini, Natália Pereira Paiva Freitas, Mariana Sartori Magnoni, Leandro Maia, Amanda Jerônimo Listoni, Marta Cristina Heckler, Mateus Jose Sudano, Marjorie Assis Golim, Fernanda da Cruz Landim-Alvarenga, Rogério Martins Amorim

**Affiliations:** 1 Departament of Veterinary Clinics, College of Veterinary Medicine and Animal Science, São Paulo State University, UNESP, Botucatu, SP, Brazil; 2 Hemocenter Division of Botucatu Medical School, São Paulo State University, UNESP, Botucatu, SP, Brazil; 3 General and Applied Biology, Botucatu Biosciences Institute, São Paulo State University, UNESP, Botucatu, SP, Brazil; 4 Laboratory of Genetics and Animal Breeding, Federal University of Pampa, Uruguaiana, RS, Brazil; 5 Department of Animal Reproduction and Veterinary Radiology, College of Veterinary Medicine and Animal Science, São Paulo State University, UNESP, Botucatu, SP, Brazil

## Abstract

**Introduction:**

Studies with mesenchymal stem cells (MSCs) are increasing due to their immunomodulatory, anti-inflammatory and tissue regenerative properties. However, there is still no agreement about the best source of equine MSCs for a bank for allogeneic therapy. The aim of this study was to evaluate the cell culture and immunophenotypic characteristics and differentiation potential of equine MSCs from bone marrow (BM-MSCs), adipose tissue (AT-MSCs) and umbilical cord (UC-MSCs) under identical *in vitro* conditions, to compare these sources for research or an allogeneic therapy cell bank.

**Methods:**

The BM-MSCs, AT-MSCs and UC-MSCs were cultured and evaluated *in vitro* for their osteogenic, adipogenic and chondrogenic differentiation potential. Additionally, MSCs were assessed for CD105, CD44, CD34, CD90 and MHC-II markers by flow cytometry, and MHC-II was also assessed by immunocytochemistry. To interpret the flow cytometry results, statistical analysis was performed using ANOVA.

**Results:**

The harvesting and culturing procedures of BM-MSCs, AT-MSCs and UC-MSCs were feasible, with an average cell growth until the third passage of 25 days for BM-MSCs, 15 days for AT-MSCs and 26 days for UC-MSCs. MSCs from all sources were able to differentiate into osteogenic (after 10 days for BM-MSCs and AT-MSCs and 15 days for UC-MSCs), adipogenic (after 8 days for BM-MSCs and AT-MSCs and 15 days for UC-MSCs) and chondrogenic (after 21 days for BM-MSCs, AT-MSCs and UC-MSCs) lineages. MSCs showed high expression of CD105, CD44 and CD90 and low or negative expression of CD34 and MHC-II. The MHC-II was not detected by immunocytochemistry techniques in any of the MSCs studied.

**Conclusions:**

The BM, AT and UC are feasible sources for harvesting equine MSCs, and their immunophenotypic and multipotency characteristics attained minimal criteria for defining MSCs. Due to the low expression of MHC-II by MSCs, all of the sources could be used in clinical trials involving allogeneic therapy in horses. However, the BM-MSCs and AT-MSCs showed fastest ‘‘in vitro’’ differentiation and AT-MSCs showed highest cell growth until third passage. These findings suggest that BM and AT may be preferable for cell banking purposes.

## Introduction

Mesenchymal stem cells (MSCs) are non-hematopoietic, multipotent progenitor cells that are easily isolated from various adult tissues. MSCs are characterized by extensive proliferative ability, as well as the ability to differentiate *in vitro* into various mesenchymal lineages in response to an appropriate stimulus. These lineages include osteoblasts, adipocytes, chondrocytes, tenocytes and myocytes [1,2]. The use of MSCs has been demonstrated in the cartilage, bone and tendon of horses [3-5]. Although controversial, MSCs can also differentiate in response to specific stimuli in germ cells of other lineages, such as neurons, glial cells and hepatocytes [6-8].

In equine species, bone marrow (BM) is one of the most studied and used sources for obtaining adult stem cells [9,10]. However, adipose tissue (AT) is also an abundant and accessible source of MSCs that can provide a large number of cells required for use in cell therapy [11,12].

Additionally, cells from the amniotic membrane [13] and umbilical cord (UC) are a promising source of MSCs because they are less immunogenic, their collection is non-invasive, and they have the potential to differentiate into neural and endothelial cells [14,15].

Equine MSCs are mainly identified by their adherence to plastic and their ability to differentiate into multiple lineages [16] because immunophenotyping in horses is hindered by the lack of specific markers, limited availability of monoclonal anti-horse antibodies [17-19] and evidence that certain markers of other species do not cross-react with equine species [11]. Therefore, several markers have been tested and used, such as the positive markers CD44, CD90 CD29 [11,15,17,20], CD105 [21-23], MHC-I [5,15,20] and the negative markers CD14 [17], CD34 [21,23], MHC-II [5,17,20,23,24], CD45 [21,24], based on minimal criteria established by the International Society for Cellular Therapy (ISCT) to define human MSCs [25] and adipose-tissue derived stromal/stem cells [26].

Evidence suggests that these cells improve regeneration and tissue function by their ability to self-renew [3], their ability to differentiate into mesodermal, neuroectodermal and endodermal lineages [6], their synthesis of growth factors and their release of anti-inflammatory and immunomodulatory cytokines [2,18,20,27].

Autologous therapy with MSCs is widely used because it does not result in any significant deleterious effects at the time of implantation or later [28], and shows anti-inflammatory and immunosuppressive effects [29]. However, treatment with autologous MSCs has limitations, such as in acute injuries, because expansion of MSCs by culturing takes 10 to 21 days [5], or in elderly patients because there is a decrease in the quantity, proliferation and differentiation potential of MSCs [30]. Nevertheless, adipose-derived nucleated cells have a short interval for isolation of an injectable uncultured cell pool (24 to 48 hours), providing distinct advantages with regard to timeliness compared with an injection of cultured MSCs from other sources [29,31].

Allogeneic treatment in horses offers advantages in acute injuries because MSCs can be injected quickly. Allogeneic treatment then eliminates the time needed for the isolation and expansion of autologous MSCs. This treatment also allows the use of a more homogeneous cell population with a proven capacity for differentiation into various lineages [5,18,31], by taking MSCs from a cell bank of horse donors [27,32]. However, a heterogeneous cell population can be more effective depending on the disease, as shown by Semon *et al*. [31], where stromal vascular fraction, which is composed of a heterogeneous mixture of cells, effectively inhibited experimental autoimmune encephalomyelitis disease progression in mice more than culture-expanded adipose derived stromal cells.

The lack of expression of the major histocompatibility complex class II (MHC-II) on the cell surface is an important immunomodulatory characteristic. This lack of expression gives MSCs the potential to escape from T-cell recognition [33], making it feasible to create a cell bank for use in allogeneic therapies [5,10,18,31].

In the present study, we assessed the differences in cell culture; immunophenotypic characterization with CD44, CD90, CD105, CD34 and MHC-II markers; and the differentiation potential of MSCs from equine bone marrow (BM-MSCs), adipose tissue (AT-MSCs) and umbilical cord tissue (UC-MSCs) aiming to compare these sources for research or an allogeneic therapy cell bank. For our knowledge, there is a lack of data comparing these characteristics (cell culture, CD expression and differentiation potential) from equine BM-, AT- and UC-MSCs at the same time and under identical *in vitro* conditions.

## Methods

### Animal ethics

All stages of this study were conducted in accordance with the Ethical Principles in Animal Experimentation and were approved by the Ethics Committee on Animal Use of São Paulo State University (UNESP) - Botucatu (Protocol 178/2011-CEUA).

### Study design

Ten clinically healthy crossbred horses of both sexes, ranging in age from 6 to 13 years old, were randomly assigned to two groups for the harvest of bone marrow (n = 5) or adipose tissue (n = 5). To obtain the umbilical cord (n = 6), two samples were collected from two births and four samples from the slaughter of horses. MSCs were cultured, and on the third passage (P3), they were assessed using immunophenotypic characterization by flow cytometry and immunocytochemistry and evaluated for their differentiation potential into three mesenchymal lineages.

### Mesenchymal stem cell collection and isolation

#### Bone marrow

The collection and isolation of BM-MSCs were performed by aspiration of the bone marrow according to the methodology described by Maia *et al*. [34] with some modifications.

Five animals (n = 5), ranging in age between 7 and 12 years, were sedated with intravenous xylazine hydrochloride 10% (0.5 mg/kg) (Sedomin, König, Avellaneda, Buenos Aires, Argentina). A local anesthetic block was then performed with 2% lidocaine hydrochloride (Xylestesin, Cristália, Sao Paulo, Brazil) in the region of the fifth sternebrae, where bone marrow cells were collected with a Komiyashiki® needle. Two syringes containing 2 mL of 1,000 IU/mL heparin (Hemofol, Cristália, São Paulo, Brazil) and 2 mL of Hanks’ Balanced Salt Solution (HBSS, Invitrogen, Grand Island, New York, USA) were used for the bone marrow collection. The samples (a total of 40 mL per animal) were centrifuged at 340 × g for 10 minutes, and the supernatant was discarded. DMEM low glucose/F12 (Invitrogen, Grand Island, New York, USA) was added to the remaining material at a ratio of 1:1, and this mixture was slowly added to Histopaque-1077 (Sigma-Aldrich Corp., St. Louis, Missouri, USA) at a ratio of 1:1, followed by further centrifugation at 340 × g for 40 minutes. After obtaining the mononuclear fraction, DMEM low glucose/F12 1:1 was added, and the sample was centrifuged twice at 340 × g for 10 minutes. The cell number was determined using the Trypan Blue exclusion method. Cells were cultured at a density of approximately 10 × 10^3^ cells/cm^2^ in 25-cm^2^ flasks (Sarstedt, Newton, North Carolina, USA) containing a culture medium consisting of 80% DMEM low glucose/F12 (1:1), 20% fetal bovine serum (Invitrogen, USA), 1% penicillin/streptomycin (Invitrogen, USA) and 1.2% amphotericin B (Invitrogen, USA) at 37°C in a humidified atmosphere containing 95% air and 5% CO_2_. The culture medium was changed every three days until a cell confluence of ≥80% was reached.

#### Adipose tissue

The collection of adipose tissue was performed in the region above the dorsal gluteal muscle (tail base) as described by Carvalho *et al*. [11]. The animals (n = 5), ranging in age between 6 and 13 years, were sedated intravenously with xylazine 10% (0.5 mg/kg) (Sedomin, König, Argentina) followed by an inverted L-block of local anesthetic using 2% lidocaine hydrochloride (Xylestesin, Cristália, Brazil).

Approximately 1 g of adipose tissue was collected and stored for 20 minutes, until arrival at the laboratory, at room temperature in a sterile 50 mL conical tube containing HBSS solution plus 2% penicillin/streptomycin.

The adipose tissue sample was washed three times with HBSS/penicillin, submitted to mechanical separation using a scalpel and anatomical forceps to approximately 0.2 to 0.3 cm size and then placed in a solution of 0.04% type 1 collagenase (Invitrogen, USA) at 37°C for 30 minutes. After this period, the solution was filtered through a 70-micrometer filter, DMEM low glucose (1:1) was added, and the solution was centrifuged twice at 340 × g for 15 minutes. A sample of cells was stained with Trypan Blue and counted using a microscope counting chamber. Cells were cultured at a density of approximately 10 × 10^3^ cells/cm^2^ in 25-cm^2^ flasks containing the culture medium described previously at 37°C in a humidified atmosphere containing 95% air and 5% CO_2_. The culture medium was changed every three days until a cell confluence of ≥80% was reached.

#### Umbilical cord

For samples from births, approximately 10 cm of umbilical cord from the fetal portion was collected immediately after delivery (n = 2) and stored in a sterile 50 mL conical tube containing HBSS plus 2% penicillin/streptomycin.

For samples from slaughterhouses (n = 4), during evisceration pregnant uteri were separated, opened and a sample of 10 cm from the umbilical cord from the fetal portion was collected. Fetuses ranging in age from 9 (n = 2) to 10 (n = 2) months were used.

Umbilical cord samples were washed three times in HBSS/penicillin and submitted to mechanical dissection to separate veins and arteries, which were discarded. The umbilical cord tissue (perivascular region) was fragmented using a scalpel and anatomical forceps to approximately 0.2 to 0.3 cm in size and then placed in a 0.04% solution of type 1 collagenase (Invitrogen, USA) at 37°C for 60 minutes. The sample was then processed as described for the adipose tissue, with the exception of a culture medium containing DMEM high glucose (Invitrogen, USA) as described by Corradetti *et al*. [35].

The trypsinization of the cells was accomplished when a confluence of ≥80% was reached. For this step, 0.25% trypsin (Invitrogen, USA) was added at 37°C for five minutes. The cell suspension was centrifuged twice at 340 × g for 10 minutes to remove the trypsin. Subsequently, the cell pellet was resuspended in culture medium, and this volume was divided into two 25-cm^2^ flasks. Cells were cultured under the same conditions as described above.

### Osteogenic, chondrogenic and adipogenic differentiation of MSCs

During the third passage (P3), BM-MSC, AT-MSC and UC-MSC samples were placed in triplicate in six-well plates (Sarstedt, USA) for osteogenic and adipogenic differentiation and were incubated at 37°C in a humidified atmosphere containing 95% air and 5% CO_2_. After reaching 80% confluence, the culture medium was removed and the differentiation media StemPro adipogenesis and StemPro osteogenesis (Invitrogen, USA) were added to the cultures. The media were changed every three days, and adipogenic differentiation was confirmed by the deposition of lipid droplets in the cytoplasm using 0.5% Oil Red O staining (Sigma-Aldrich Corp., USA). The osteogenic differentiation was confirmed by positive staining of the extracellular calcium matrix using 2% Alizarin Red S staining (Sigma-Aldrich Corp., USA).

For chondrogenic differentiation, a pellet of MSCs was cultured in a Falcon tube and incubated at 37°C in a humidified atmosphere containing 95% air and 5% CO_2_. After two days, the culture medium was removed and the differentiation medium StemPro chondrogenesis (Invitrogen, USA) was added and changed every three days. To confirm chondrogenic differentiation, pellets were stained with Alcian Blue (pH = 2.5) and toluidine blue (pH = 1) to identify proteoglycans.

### MSC characterization

#### Flow cytometry

Immunophenotypic analysis of BM-MSCs, AT-MSCs and UC-MSCs was performed at P3 with the FACS Calibur flow cytometer (BD, Franklin Lakes, NJ, USA) using the following antibodies: mouse anti-rat CD90-FITC (clone OX7, Caltag Laboratories, Burlingame, California, USA), mouse anti-human CD34-FITC (clone 581, BD, USA), mouse anti-human CD105-FITC (clone SN6, Abcam, San Francisco, California, USA), mouse anti-horse CD44 (clone CVS18, AbD Serotec, Kidlington, Oxfordshire, UK) and mouse anti-horse MHC class II monomorphic (clone CVS20, AbD Serotec, UK). For the unconjugated primary markers, the secondary antibody goat anti-mouse IgG-FITC (AbD Serotec, UK) was used. The protocols used were those described by the manufacturers.

#### Immunocytochemistry for MHC-II

Samples at P3 were plated in 24-well plates (Sarstedt, USA). After 80% confluence was reached, the cells were fixed and permeabilized with Cytofix/Cytoperm™ (BD, USA), and then an endogenous peroxidase block was conducted for 20 minutes. After this period, the samples were incubated for one hour in a 3% milk powder solution for nonspecific protein blocking. Then, the primary antibody mouse anti-horse MHC class II monomorphic (clone CVS20, Abd Serotec, UK) and negative control IgG anti-mouse (Dako Cytomation, Glostrup, Denmark) were incubated in the dark for 18 hours at 4°C. The primary antibodies were detected by incubating with the polymer system HiDef HRP mouse/rabbit Polymer Detection System (Cell Marque, Rocklin, CA, USA). The development reaction was conducted using the chromogenic substrate DAB solution (Dako Cytomation, Denmark) for five minutes, followed by counterstaining with Harris Hematoxylin (Merck, Rockland, Massachusetts, USA) for one minute. The evaluation of the reaction was performed using an inverted light microscope (Leica Microsystems, Wetzlar, Germany).

The positive control was performed for MHC-II antibody using a horse muscle tissue sample.

### Statistical analysis

For the statistical analysis, the binomial dependent variables (positive marker percentage of CD105, CD90, CD44, CD34 and MHC-II) were evaluated with an ANOVA using the General Linear Model (GLM) procedure with the SAS statistical software package (SAS, Inst., Inc., Cary, NC, USA). Sources of variation in the model included cellular source (UC, BM or AT) and animals, which were considered as fixed and random effects, respectively. The arcsine transformation was applied to the percentage of data to improve normality. If the ANOVA was significant, means were separated using the least significant difference (LSD). The data are reported as the least-squares means ± SEM. For all analyses, a significance level of 5% was used.

## Results

### Cell culture

All of the MSCs cultured from BM, AT and UC adhered to the flasks in the first days of culture. Adhesion of MSCs to the plastic flask was observed within 48 hours for BM-MSCs, 32 hours for AT-MSCs and 48 hours for UC-MSCs.

**Figure 1:**
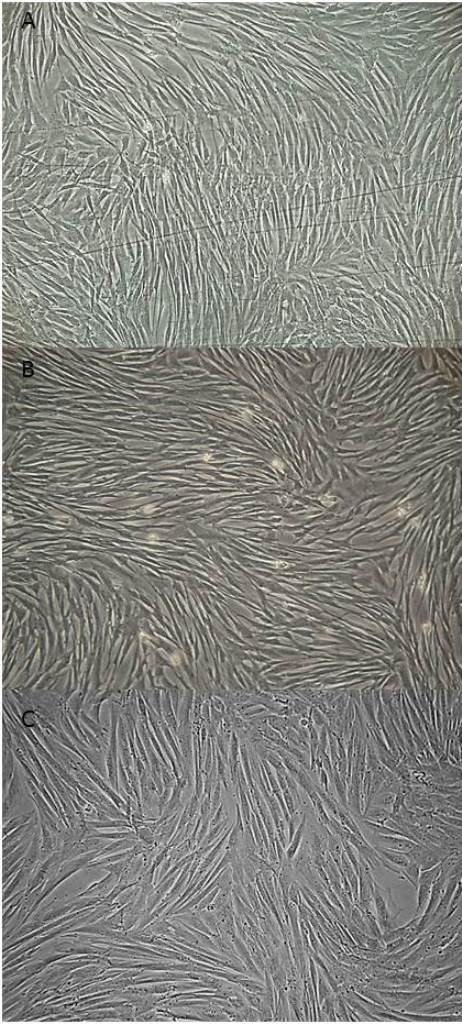
**MSCs from BM, AT and UC during cell culture showing ≥80% confluence.** MSCs from BM **(A)**, AT **(B)** and UC **(C)** with approximately 80% confluence after 11, 7 and 15 days of culture, respectively. **(A)** and **(B)** 100x magnification and **(C)** 200x magnification. AT, adipose tissue; BM, bone marrow; MSCs, mesenchymal stem cells; UC, umbilical cord.

Early fibroblastoid morphology in MSCs was visualized with an average of 4.5 ± 0.70 days of culture for MSCs from BM, 3.5 ± 2.12 days for MSCs from AT and 4.8 ± 1.30 days for MSCs from UC.The time to reach approximately 80% cell confluence differed between the samples: 11 days for BM-MSCs, 7.3 ± 1.52 days for AT-MSCs and 15.25 ± 6.65 days for UC-MSCs (Figure 1). After the first passage, 80% confluence was achieved with an average of 5.2 ± 1.64 days for BM-MSCs, 3.2 ± 0.44 days for AT-MSCs and 6.16 ± 2.40 days for UC-MSCs.

The time from primary culture until the third passage, when characterization and cell differentiation was performed, was an average of 25 ± 3.93 days for BM-MSCs, 15.5 ± 0.70 days for AT-MSCs and 26.75 ± 7.22 days for UC-MSCs.

### Potential of differentiation into mesodermal lineages

**Figure 2:**
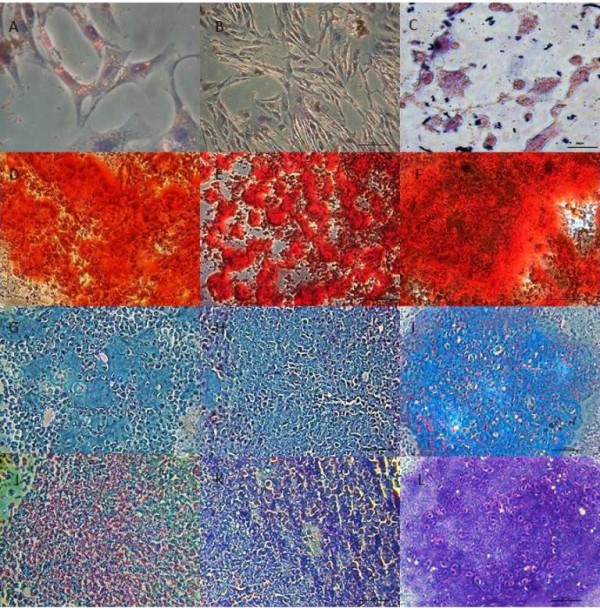
**Differentiation potential of MSCs from equine BM, AT and UC.** Differentiation of MSCs from BM **(A, D, G, J)**, AT **(B, E, H, K)** and UC **(C, F, I, L)** into three mesenchymal lineages during the third passage. **(A-C)** MSCs showing intracytoplasmic lipid droplets confirming the adipogenic lineage. **(D-F)** MSCs stained with Alizarin Red showing matrix calcium formation. **(G-L)** MSCs after chondrogenic differentiation stained with Alcian Blue **(G-I)** and toluidine blue **(J-L)** showing hyaline matrix. 100x **(D-L)**, 200x **(B)** and 400x **(A, C)** magnification. AT, adipose tissue; BM, bone marrow; MSCs, mesenchymal stem cells; UC, umbilical cord.

The *in vitro* differentiation potential of BM- and AT-MSCs for osteogenic and adipogenic lineages was shown after the 10th and 8th days, respectively. The UC-MSCs did not differentiate in this time interval, requiring new differentiation repetitions. The differentiation into osteocytes and adipocytes was observed only after 15 days in these cells.The osteogenic differentiation was confirmed by positive staining of the calcium matrix by Alizarin Red dye. Additionally, a change from fibroblastoid morphology to a predominantly polygonal morphology in a large proportion of the cells was observed (Figure 2). The differentiation into adipose tissue was confirmed by the deposition of lipid droplets into the cytoplasm, as visualized by Oil Red staining (Figure 2).The chondrogenic differentiation occurred after 21 days for MSCs from BM, AT and UC and was confirmed by the deposition of a hyaline matrix rich in proteoglycans. The Alcian Blue (blue areas) and toluidine blue staining (metachromatic pink areas) (Figure 2) were performed, which identified an extracellular matrix rich in proteoglycans.

### Immunophenotypic characterization

**Figure 3:**
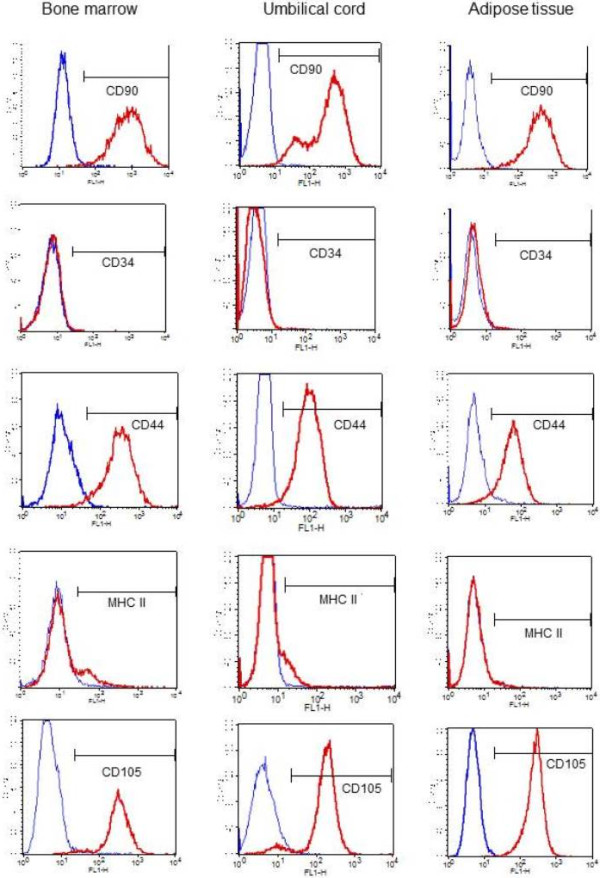
**Expression of cell surface markers by flow cytometry of MSCs from BM, AT and UC.** Histograms representing the profile of BM-MSC, AT-MSC and UC-MSC samples analyzed using flow cytometry during the third passage, evaluating the cell surface markers CD90, CD44, CD105, CD34 and MHC-II. MSCs were positive for CD90, CD44 and CD105, were negative for CD34 and showed low expression of MHC-II. AT, adipose tissue; BM, bone marrow; MSCs, mesenchymal stem cells; UC, umbilical cord.

**Figure 4:**
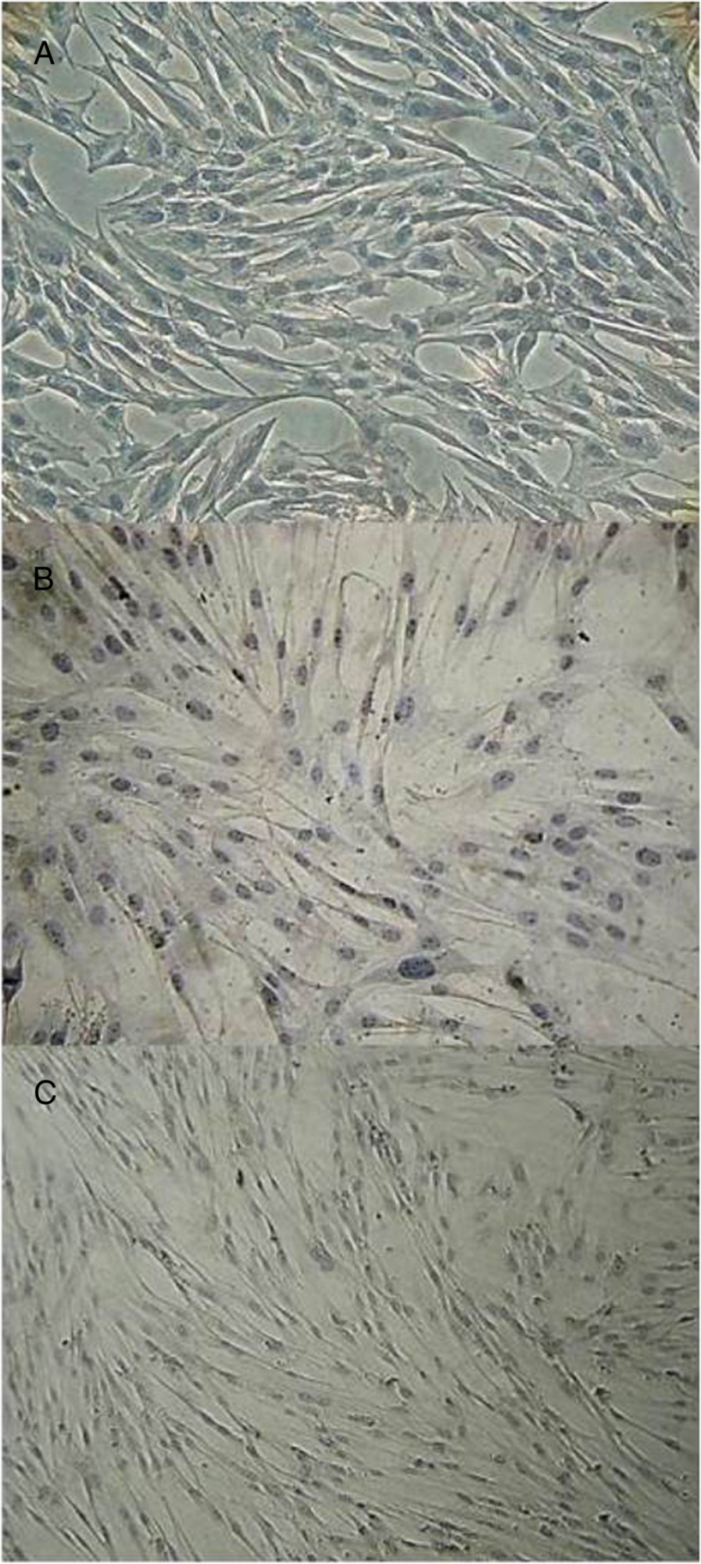
**Evaluation of MHC-II expression in MSCs from BM, AT and UC using the immunocytochemistry technique.** Immunocytochemistry of MSCs from equine BM **(A)**, AT **(B)** and UC **(C)** during the third passage to evaluate the expression of the MHC-II cell surface marker. MSCs from the sources analyzed did not express MHC-II. 200x **(A, B)** and 100x **(C)** magnification. AT, adipose tissue; BM, bone marrow; MSCs, mesenchymal stem cells; UC, umbilical cord.

Immunophenotypic analysis at P3 of BM-MSCs, AT-MSCs and UC-MSCs by flow cytometry revealed MSCs with a high expression of CD90, CD105 and CD44 markers and a low or absent expression of CD34 and MHC II markers (Figure 3). A minimum of 10,000 cells were used for flow cytometry evaluation. The average percentage with standard deviation for each marker from the different sources of MSCs can be observed in Table 1.MSCs from BM, AT and UC showed no expression of MHC-II as assessed by immunocytochemistry techniques (Figure 4).

**Table 1:** Percentage means of the immunophenotypic characterization of BM-MSCs, AT-MSCs and UC-MSCs by flow cytometry

**Source**	**Marker**				
	**CD105**	**CD90**	**CD44**	**CD34**	**MHC-II**
**UC**	94.2 ± 2.1	67.7 ± 6.5^a^	95.7 ± 1.5	0.20 ± 0.3^a^	5.9 ± 1.8
**BM**	97.7 ± 2.3	98.2 ± 7.1^b^	96.2 ± 1.6	0.28 ± 0.3^a^	6.8 ± 2.0
**AT**	94.2 ± 2.3	99.2 ± 7.1^b^	92.4 ± 1.6	1.21 ± 0.3^b^	3.8 ± 2.0
*P*-value	0.5442	0.0033	0.1994	0.0197	0.4896

Percentage means of MSCs from BM, AT and UC during the third passage for the CD90, CD105, CD44, CD34 and MHC-II markers by flow cytometry. AT, adipose tissue; BM, bone marrow; MSCs, mesenchymal stem cells; UC, umbilical cord.

^a,b^Overrides different letters in the same column that differ (*P* <0.05).

## Discussion

Several sources have been studied for obtaining equine MSCs, such as bone marrow [34], adipose tissue [11], umbilical cord blood [8], umbilical cord tissue [36], amniotic membrane [13], peripheral blood [37], tendon [27] and amniotic fluid [23]. Bone marrow and adipose tissue are the most studied sources [9,12,21,38], but recent studies are using cells from other sources, such as fetal tissues [15,31,39], because these samples can be obtained non-invasively and they are easily accessible, abundant and easy to collect [22,23].

Bone marrow puncture requires a qualified professional, can be painful and is an invasive technique with the risk of thoracic puncture [13,40], but it is a quick method of harvest and causes less site injury than adipose tissue harvest [28], although there are minimally invasive methods described to harvest adipose tissue, such as liposuction [41].

Adipose tissue has advantages over BM, such as an abundance in the body, ease of collection and the ability to culture a larger number of cells with better quality [9,42]. However, it can be difficult to collect AT samples from lean horses [29,40].

In humans, fetal cells have advantages over adult cells because they can proliferate faster *in vitro*, have low levels of histocompatibility antigens and can survive at lower oxygen tensions, making them more resistant to hypoxia during transplantation [14]. However, as observed in our study, equine fetal cells do not multiply as rapidly as those in adipose tissue [23].

Stem cells from the umbilical cord (UC) of horses can be isolated from umbilical cord tissue (UCT), Wharton’s jelly or from the umbilical cord blood (UCB). The technique for collection is minimally invasive and can be performed without harm to the foal or mare [36,43].

In the present study, BM, AT and UC were used as sources for obtaining equine MSCs, and the MSCs’ expression of surface markers and their differentiation potential into osteogenic, adipogenic and chondrogenic lineages were evaluated to compare these sources for an allogeneic therapy cell bank. All stages of this study were performed during the third passage because the cells reached homogeneous culture at this point, as observed in other studies [21,44,45].

MSC differentiation into osteogenic, adipogenic and chondrogenic mesodermal lineages is an important feature that should always be used to verify the multipotentiality of MSCs [22], so in this study, BM-MSCs, AT-MSCs and UC-MSCs underwent differentiation into mesodermal lineages to prove the differentiation potential of MSCs from each of the sources studied as postulated by Dominici *et al*. [25].

The BM-MSCs and AT-MSCs differentiated within 10 days into the osteogenic lineage and within 8 days into the adipogenic lineage. This time period is in agreement with other studies with MSCs from BM and AT, where they differentiated within 7 to 15 days into the adipogenic lineage and within 7 to 18 days into the osteogenic lineage [21,27,46].

The UC-MSCs used in this study did not differentiate into these lineages in the same time period; the osteogenic and adipogenic differentiation occurred only after 15 days. This longer time period required for differentiation has been reported in other studies, where osteogenic differentiation occurred in 14 to 21 days [22,23] and adipogenic differentiation within 25 days [23]. However, Corradetti *et al*. [35] reported an osteogenic differentiation of UCT in just 10 days. Toupadakis *et al*. [40] reported that the addition of 20% fetal calf serum (FCS) induces faster and more efficient osteogenic differentiation.

Despite the difference in the time required for cell differentiation among the sources, there were no differences in the differentiation potential of BM-MSCs, AT-MSCs and UC-MSCs because all cells were able to differentiate into the three mesodermal lineages. Nevertheless, studies have reported that osteogenic differentiation of MSCs is better in BM-MSCs and that chondrogenic differentiation can be better in UC-MSCs [23,27] or BM-MSCs [40,46]. Moreover, MSCs from an equine umbilical cord matrix may be able to differentiate into neuronal cells in addition to these three lineages [22].

Due to the lack of specific equine antibodies and the low reactivity of markers from other species to equine species [11,17], different markers have been tested and used. In the present study, the CD90, CD34, CD44, CD105 and MHC-II markers were used, taking into account some of the criteria established by the International Society for Cellular Therapy for the characterization of human MSCs [25] and adipose tissue-derived stromal/stem cells [26], as well as other studies with equine MSCs that already used some of these markers [20,21,23,24,45,47]. Additionally, it has been shown that human AT-MSCs can be distinguished from BM-MSCs by their positivity for CD36 and negativity for CD106 [26], but it is not determined in equine MSCs.

The MSCs from BM, AT and UC under the culture conditions utilized showed high positivity for CD90, CD44 and CD105, were negative for CD34 and showed low expression of MHC-II.

Several studies with equine MSCs also showed high expression of CD90 (82 to 93%), CD44 (79 to 98%) and CD105 (78 to 93%) [16,20,23,44,48]. However, Xie *et al*. [45] demonstrated the lowest expression of CD 105 (64%) in BM samples, and De Schauwer *et al*. [24] observed a large variation of CD105 expression (0, 1 to 20%) among UCB samples, and another study with UCT [35] reported negative expression of CD105, possibly because the antibody did not work for the cells studied.

In the present study, UC-MSCs showed lower expression of the CD90 marker (mean 67.7%) when compared with the BM-MSCs and AT-MSCs, unlike other studies [43,44,49] where the expression was higher. However, a study [50] with amniotic fluid demonstrated that the CD90 marker decreases as pregnancy develops. This inverse relationship may explain the findings in this present study because the samples collected in the slaughterhouse were from the final months of gestation (9 to 10 months).

Studies using AT-MSCs [12,21] reported increased CD34 expression compared to BM-MSCs; however, this expression was higher than that obtained in the present study (mean 1.1%).

The differences between animals may be due to laboratorial procedures and the biological differences between the donors because the species, age, gender and site of collection may influence the number, phenotype and *in vitro* biological characteristics of the MSCs [16].

The MHC-II expression in BM-MSCs, AT-MSCs and UC-MSCs did not show significant differences, revealing low expression for all samples. This result is similar to a study of MSCs from UCB, which also showed low expression of MHC-II (8%) [24]. However, AT-MSCs had the lowest expression (3.8% on average) of MHC-II, which would result in a reduced risk of immune reaction against allogeneic therapy. In several other studies, the expression of MHC-II was negative by flow cytometry [20,22] and RT-PCR [23,35] techniques. Expression was observed in the present study only by the immunocytochemistry technique. Therefore, more studies should be performed to better characterize the low expression or lack of expression of MHC-II in several cellular sources from horses.

The bone marrow and adipose tissues were the best cell sources for obtaining MSCs under the *in vitro* conditions used because they differentiated in a faster time and showed higher cell growth than umbilical cord. Koerner *et al*. [37] reported that BM is the most valuable and reliable source of MSCs for cell therapy in the horse when compared with peripheral blood. *In vitro* studies demonstrated that BM-MSCs have better cell differentiation potential than AT-MSCs, suggesting that they are a superior source of cells for the musculoskeletal regeneration of horses, but this difference has not yet been shown *in vivo*[9]. Additionally, when comparing BM with UCB [14], MSCs from BM have more mesenchymal progenitor cells. The proliferation and differentiation capacities of BM-MSCs are inversely proportional to the age of the donors and the number of passages in cell culture [13,21].

In our study, AT-MSCs showed the highest cell growth until the third passage, corroborating with studies comparing BM and AT as sources of MSCs and reporting that AT has a larger amount of MSCs with a higher potential for proliferation [18,27,38,42], which would make adipose tissue an advantageous source for allogeneic bank creation. Even after cryopreservation, they retain their proliferative potential and differentiation capacity [51].

On the other hand, studies with MSCs from UCB [14,30] describe some advantages over BM cells: they are less immunogenic, they cause less graft versus host disease, their collection is noninvasive, they have a higher proliferative rate, they have a higher number of cells per volume collected, and their ability to be cryopreserved generates a ready-to-use product.

Despite our findings, further studies are required to assess changes in the differentiation potential of BM-MSCs, AT-MSCs and UC-MSCs at the higher passages, as well as to elucidate biological mechanisms involved in therapeutic potential and senescence comparing several sources for equine MSCs. There is still no consensus on what would be the best source for obtaining these cells for cell bank and clinical purposes due to the conflicting results among studies.

## Conclusions

Equine BM, AT and UC are viable sources for obtaining MSCs based on the principles established by the ISCT, as this study confirmed the immunophenotypic and multipotentiality characteristics of these cells. Based on the low expression of MHC-II, BM-MSCs, AT-MSCs and UC-MSCs could be used in clinical trials involving allogeneic therapy in horses. Under the experimental conditions of this study, the BM-MSCs and AT-MSCs showed fastest *in vitro* differentiation and AT-MSCs showed highest cell growth until the third passage. These findings suggest that BM and AT may be preferable for cell banking purposes.

## Abbreviations

AT: Adipose tissue; AT-MSCs: Mesenchymal stem cells from adipose tissue; BM: Bone marrow; BM-MSCs: Mesenchymal stem cells from bone marrow; DMEM: Dulbecco’s Modified Eagle Medium; HBSS: Hanks’ Balanced Salt Solution; ISCT: International Society for Cellular Therapy; LSD: Least significant difference; MHC-II: Major histocompatibility complex class II; MSCs: Mesenchymal stem cells; P3: Third passage; UC: Umbilical cord; UCB: Umbilical cord blood; UC-MSCs: Mesenchymal stem cells from umbilical cord; UCT: Umbilical cord tissue.

## Competing interests

The authors declare that they have no competing interests.

## Authors’ contributions

DJB was responsible for the conception and design of the study, data collection and analysis of BM, AT and UC-MSCs, labelling of MSCs for flow cytometry, immunocytochemistry and cell differentiation, manuscript writing and giving final approval of the manuscript. NPPF assisted with collections of AT, participated in the isolation and cultivation of AT-MSCs and assisted in the preparation of MSCs for flow cytometry. MSM performed the analysis and interpretation of data from flow cytometry of AT-MSCs and UC-MSCs. LM participated in the collection, isolation and culture of BM-MSCs and assisted in immunocytochemistry and cellular differentiation experiments. AJL participated in the isolation and culture of BM-MSCs and assisted with the cell differentiation staining. MCH participated in the collections of BM and assisted in the isolation and culture of UC-MSCs. MJS performed the statistical analysis and interpreted the data. MAG performed the analysis and interpretation of BM-MSC flow cytometry data. FCLA was responsible for supervision of the isolation and culture of MSCs in the laboratory. RMA contributed to the conception and coordination of the study, participated in the collection of samples from BM, manuscript writing and final approval of the manuscript. All authors read and approved the final manuscript.
